# Impact of glucose and propionic acid on even and odd chain fatty acid profiles of oleaginous yeasts

**DOI:** 10.1186/s12866-025-03788-w

**Published:** 2025-02-18

**Authors:** Veronica Bonzanini, Majid Haddad Momeni, Kim Olofsson, Lisbeth Olsson, Cecilia Geijer

**Affiliations:** 1 Department of Life Sciences, Division of Industrial Biotechnology, Chalmers University of Technology, Chalmersplatsen 4, Gothenburg, 412 96 Sweden; 2 AAK AB, Pulpetgatan 20, Malmö, 215 37 Sweden

**Keywords:** Odd chain fatty acid (OCFA), Even chain fatty acid (ECFA), Short chain fatty acid (SCFA), Non-conventional yeast, Propionic acid tolerance, Fatty acids methyl ester, *Rhodotorula toruloides*, *Yarrowia lipolytica*, *Cutaneotrichosporon oleaginosus*

## Abstract

**Background:**

Odd chain fatty acids (OCFAs) are gaining attention for their valuable medical and nutritional applications. Microbial fermentation offers a sustainable and environmentally friendly alternative for OCFA production compared to traditional extraction or chemical synthesis methods. To achieve an economically feasible OCFA production process, it is essential to identify and develop microbial cell factories capable of producing OCFAs with high titers and yields.

**Results:**

We selected 19 yeast species, including both oleaginous yeasts and representatives from the *Ascomycota* and *Basidiomycota* phyla, based on their known or potential ability to produce OCFAs. These species were screened under various growth conditions to evaluate their OCFA production potential. In glucose-based, nitrogen-limited media, the strains produced fatty acids to varying extents, with OCFAs comprising 0.5–5% of the total fatty acids. When using the OCFAs precursor propionic acid as the sole carbon source, only eight strains exhibited growth, with tolerance to propionic acid concentrations between 5 and 29 g/L. The strains also displayed varying efficiencies in converting propionic acid into fatty acids, yielding between 0.16 and 1.22 g/L of fatty acids, with OCFAs constituting 37–89% of total fatty acids. Among the top performing strains, *Cutaneotrichosporon oleaginosus* produced the highest OCFA titers and yields (0.94 g/L, 0.07 g/g), *Yarrowia lipolytica* demonstrated superior growth rates even at elevated propionic acid concentrations, and *Rhodotorula toruloides* achieved the highest proportion of OCFAs relative to total fatty acids (89%).

**Conclusions:**

Our findings highlight the diverse capacities of the selected yeast species for OCFA production, identifying several promising strains for further optimization as microbial cell factories in sustainable OCFA production processes.

**Graphical Abstract:**

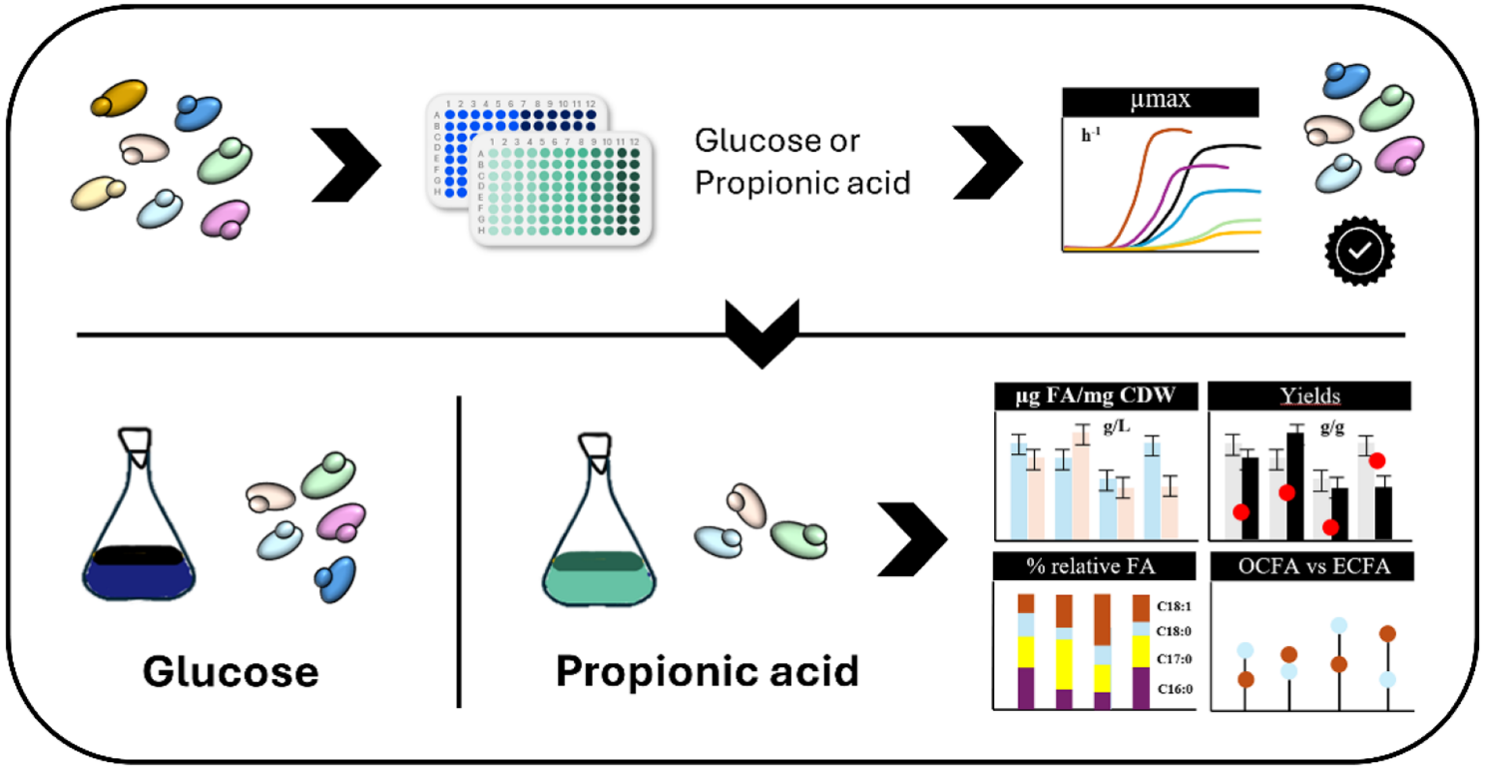

**Supplementary Information:**

The online version contains supplementary material available at 10.1186/s12866-025-03788-w.

## Background

Odd chain fatty acids (OCFAs), categorized by hydrocarbon chains with an odd number of carbons, are gaining increasing recognition for their medical and nutritional applications. The primary OCFAs found in human plasma phospholipids include pentadecanoic acid (C15:0), heptadecanoic acid (C17:0), heptadecenoic acid (C17:1) and tricosanoic acid (C23:0) [1, 2]. Among these, C15:0 and C17:0 have been the most extensively studied in clinical research. An inadequate dietary intake of OCFAs has been shown to be associated with higher risks of developing diseases like adiposity, chronic inflammation, cardiovascular disease, type 2 diabetes, and certain types of cancer [3–7]. OCFAs can also be used as building blocks for the synthesis of very long chain fatty acids (> 22 carbons) with an odd number of carbons [8], which are used to generate glycosphingolipids in the brain that are important molecules for mediating signal transduction and cell adhesion [9]. Furthermore, cis-9-heptadecenoic acid (C17:1) is used to treat conditions such as psoriasis and allergies [10]. In addition to the pharmacological and nutritional importance, OCFAs also serve as crucial precursors in the manufacturing of pesticides, fragrances, cosmetics, coatings, and biofuels [11].

Current OCFA production methods, including extraction from plant-based materials and chemical synthesis, are associated with high costs, low efficiency, and environmental concerns. In contrast, microbial fermentation offers a promising and sustainable alternative for OCFA production. Among potential microbial cell factories, oleaginous yeast species are particularly interesting due to their capability to accumulate over 20% of their total dry weight in lipids, predominantly stored as triacylglycerols (TAGs) in lipid bodies [12]. OCFAs, however, typically constitute only a minor fraction of the total fatty acids produced by these yeasts. By applying specific growth conditions, such as addition of odd chain precursors, it is possible to significantly enhance their OCFA production [13, 14]. To date, OCFAs research has focused on engineering *Yarrowia lipolytica* for the production of OCFAs from glucose or odd chain precursors [15–19], although other yeasts such as *Cutaneotrichosporon cutaneum*, *Cutaneotrichosporon oleaginosus* (also known as *Cryptococcus curvatus)*, *Rhodotorula glutinis*, *Candida spp.*, *Kluyveromyces polysporus*, *Torulaspora delbrueckii*, and *Rhodotorula toruloides* also demonstrate the capacity to produce OCFAs [13, 14, 20, 21]. To establish cost-competitive production processes for OCFAs, it is crucial to deepen our understanding of yeast metabolism and to develop specialized production strains and processes tailored for this purpose.

**Fig. 1:**
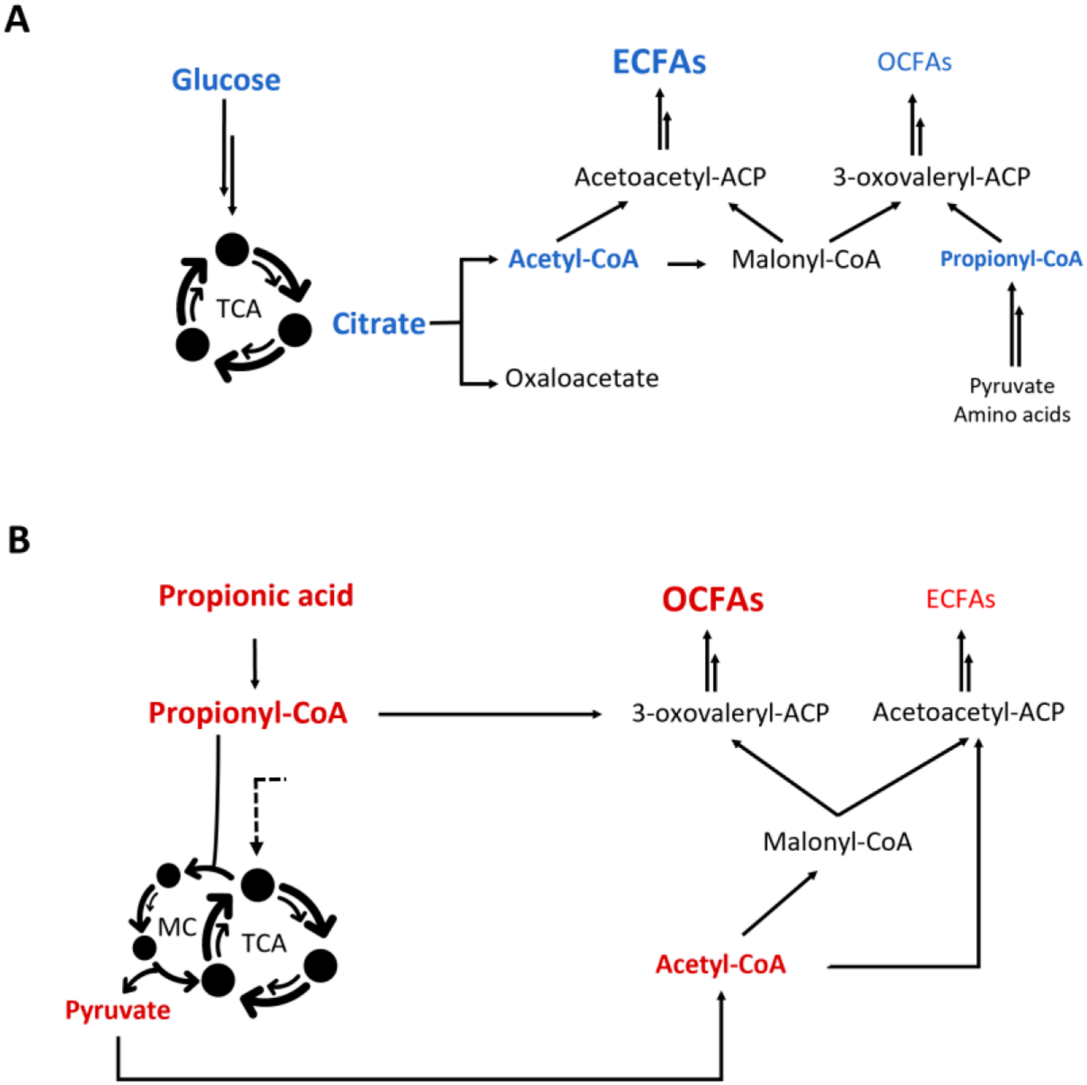
General overview of fatty acid metabolism in yeast under nitrogen limitation. **A**) Upon yeast’s growth in glucose-based medium under nitrogen starvation, the metabolism predominantly results in ECFAs, with only a negligible production of OCFAs; **B**) In a propionic acid-based medium, yeast produces elevated amounts of OCFAs. Additionally, the synthesis of ECFAs persists, albeit to a much lower extent with respect to glucose-based growth conditions. Double arrows represent multiple reactions; dotted arrows represent unknown pathways; MC, methyl citrate cycle; TCA, tricarboxylic acids cycle; TAGs, triacylglycerols; OCFAs, odd chain fatty acids; ECFAs, even chain fatty acids

*De novo* fatty acid biosynthesis in oleaginous yeasts using sugar-based substrates is initiated when starvation of nitrogen and/or phosphate halts biomass formation and redirects metabolism towards lipid accumulation [22, 23]. Under nitrogen starvation conditions, the TCA cycle slows down, leading to citrate accumulation in the mitochondrion. To prevent toxicity, citrate is transported to the cytoplasm and cleaved to generate acetyl-CoA and oxaloacetate [24–26]. Acetyl-CoA then enters the fatty acid elongation cycle, where two-carbon units from malonyl-CoA are sequentially added to the growing acyl chain, leading to the formation of even chain fatty acids (ECFAs) (Fig. 1A). To produce OCFAs, acetyl-CoA is replaced by propionyl-CoA as the precursor. Numerous metabolic pathways can generate propionyl-CoA from sugar catabolism, including the 2-oxobutyrate pathway, the methyl malonyl-CoA pathway, and the amino acid degradation pathway [11]. While yeasts usually produce no more than 3% of OCFAs on total fatty acids [19], *Blastobotrys adeninivorans* and *Wickerhamomyces anomalus* have been documented to produce up to 30% OCFAs of the total fatty acid content when cultivated in nutrient-rich media [27]. Furthermore, only a small fraction of all existing oleaginous yeasts has been characterized, and due to the largely unexplored potential of microbial OCFA production, it remains to be determined whether additional yeasts can produce higher levels of OCFAs under sugar-based growth conditions.

Besides a few pioneering studies on sugar-based OCFA production [16, 28], most studies concentrate on supplementing the yeast cultivation medium with odd chain precursors such as 1-propanol, or more commonly the short chain fatty acid (SCFA) propionic acid (PA) [13, 14, 21, 24, 29]. Although the metabolic pathways involved in PA uptake and assimilation in yeasts remain largely unexplored, it is known that PA can be converted intracellularly to the OCFAs precursor propionyl-CoA (Fig. 1B). As high concentrations of PA are known to lower the intracellular pH and inhibit metabolism and growth [30], yeasts that can tolerate high concentrations of PA have potential to become new workhorses for OCFA production. PA can be produced in a sustainable manner through microbial anaerobic fermentation of food waste, agricultural residues or lignocellulosic biomasses. This fermentation process involves the complex degradation of organic matter, ultimately leading to biogas production. During the hydrolytic and acidogenic stages of anaerobic fermentation, PA is generated alongside other short-chain fatty acids (SCFAs) such as acetic acid, butyric acid, valeric acid, and caproic acid [24].

In the present study, our aim was to gain deeper understanding of the fatty acid profiles and production capacities of selected yeast species, using either glucose or PA, as carbon source. Based on our findings, we sought to identify strains with potential as future hosts for OCFA production.

## Materials and methods

### Yeast strains, activation and storage

All yeast strains characterized in this study were obtained from culture collections. *Cutanetrichosporon oleaginosus* ATCC 20,509 was acquired from the American Type Culture Collection, Virginia, USA (ATCC), while *Rhodotorula toruloides* CBS 14 and *Yarrowia lipolytica* W29 (CBS 7504) were procured from Westerdijk Fungal Biodiversity Institute, Utrecht, Netherlands (CBS). *Barnettozyma californica* Y-1680, *Blastobotrys adeninivorans* Y-17,692, *Blastobotrys raffinosifermentans* Y-27,150, *Cutaneotrichosporon cutaneum* Y-2526, *Debaryomyces hansenii* Y-7426, *Lipomyces tetrasporus* Y-11,562, *Metschnikowia pulcherrima* Y-711, *Pichia occidentalis* Y-1732, *Rhodosporidiobolus fluvialis* Y-12,922, *Rhodotorula mucilaginosa* Y-1593, *Saturnispora silvae* Y-6725, *Schwanniomyces occidentalis* Y-2470, *Starmerella bombicola* Y-17,069, *Torulaspora delbrueckii* Y-2229, *Wickerhamomyces anomalus* Y-17,698 and *Zygotorulaspora florentina* Y-1560 all came from National Center for Agricultural Utilization Research, Illinois, USA (NRRL).

Activation of the strains was conducted in YPD (yeast extract 10 g/L, peptone 20 g/L, dextrose 20 g/L) or PD (potato extract 4 g/L, glucose 20 g/L) medium following the instructions provided by the respective culture collections. Glycerol stocks were prepared with a final concentration of 25% v/v glycerol in sterile 1 mL cryotubes and 96-well plates and stored at -80 ºC.

### Cultivation media

To promote growth, the pre-culture medium contained a low molar carbon to nitrogen ratio (C/N ratio 9) and consisted of 1.7 g/L YNB (Yeast Nitrogen Base) without amino acids and ammonium sulfate (filter sterilized), 20 g/L glucose and 5 g/L (NH4)2SO_4_. Also 50 mM phosphate buffer derived from 28.2 g/L Na_2_HPO_4_ and 11.45 g/L KH_2_PO_4_ was included to maintain the pH at 6.8. The yeasts were inoculated directly from glycerol stocks into sterile culture tubes with 3–5 mL of pre-culture medium and grown until reaching stationary phase (48–72 h) at 30 ºC, 200 rpm. *Z. florentina* was pre-cultured at its optimal temperature (25 ºC) to favor biomass formation.

The glucose-based and PA-based growth media contained the same components as the pre-culture medium, maintaining a molar C/N ratio of 9. Similarly, the glucose-based and PA-based fatty acid accumulation media matched the pre-culture composition but with an increased molar C/N ratio of 50. In all setups, the ammonium sulfate concentration was adjusted to achieve the desired C/N ratio based on available carbon. For lipid production in flasks, the glucose-based medium contained 20 g/L of glucose, while the PA-based medium included 15 g/L of PA.

To maintain a pH of 6.8 in the PA-based media with concentrations $\:\ge\:$15 g/L, potassium salt in the phosphate buffer was here replaced with the more basic version (K_2_HPO_4_), holding the same molarity, while the final concentration of the phosphate buffer in the medium was increased to 100 mM.

### Microtiter growth profiles cultivations

To assess the growth differences in glucose at molar C/N ratios of 9 and 50 and determine the PA-tolerance, screens in 96-well plates were conducted. The PA tolerance-growth test was carried out by exposing yeast strains to varying concentrations of PA at C/N ratio 9 with six different concentrations of PA: 5, 10, 15, 19, 24 and 29 g/L. Additionally, growth experiments where 2 g/L glucose was combined with 5 and 10 g/L of PA were performed. Growth was monitored in real-time using the Growth Profiler 960 by Enzyscreen (Heemstede, Netherlands), with the OD equivalent unit *green values* serving as the output from image analysis. The Growth Profiler utilizes image analysis, capturing pictures from the transparent bottom of the 96-well plate to measure cell density based on 30 min intervals. The green values depict yeast cell growth based on pixel counts from multiple images. To prevent evaporation, the external perimetral wells in the plates were filled with water. In all conditions the yeasts were inoculated at OD 0.1 and were grown at 30 ºC and 250 rpm for about 4–5 days in glucose-based medium and 6 days in PA-based medium. Each yeast strain was grown as biological triplicates under every condition, and means and standard deviations were calculated. The maximum specific growth rates were calculated using the *all_spline* function in R.

### Shake-flask cultivations for fatty acid production

To determine growth and fatty acid production, the strains were cultivated in 250 mL flasks containing 85 mL of medium starting from OD 0.1 and incubated in a Cell Growth Quantifier (CGQ) (Sbi– Scientific Bioprocessing, Pennsylvania, USA), at 200 rpm and 30 °C. The growth profiles were monitored in real-time using OD equivalent unit *backscatter light* as output. Growth and production parameters including cell dry weight (CDW g/L), substrate consumption (g/L) and fatty acids (µg fatty acid/mg CDW; g/L) were measured after 115 and 188 h in the presence of glucose and PA, respectively. Sampling times were chosen for strains to maximize carbon source consumption and reach the stationary phase, promoting lipid production and accumulation. Each strain was cultivated in two biological replicates, and measurements of fatty acids and CDW were performed in at least three technical replicates. Means and standard deviations were calculated for analysis.

### Determination of cell dry mass

CDWs were determined by collecting measured volumes of culture into pre-weighed glass tubes. The cells were washed twice with Milli-Q water, centrifuged at 3000 rpm for 5 min each time, and the supernatants were discarded. The cell pellets were then freeze-dried overnight using a freeze dryer (-50 °C). The following day, the tubes were weighed again, and CDWs were calculated in g/L.

### HPLC to measure residual substrate

In the shake-flask cultivations, residual substrate concentrations (g/L) of glucose or PA were measured by collecting the supernatant after centrifugation, followed by filtration (0.22 μm). Samples were frozen until analysis. The residual substrate concentrations were determined using HPLC (Jasco, Tokyo, Japan). Separation was performed using the Rezex ROA- Organic Acids H + column (Phenomenex, Part No. 00 H-0138-K0, 300 × 7.8 mm), with a mobile phase consisting of 5 mM H_2_SO_4_ and a matrix of sulfonated styrene divinyl benzene. The method employed had a duration of 18 min, with the oven temperature set at 80 °C and a flow rate of 0.8 mL/min. Refractive index (RI-4030/4035 Jasco) detection was utilized. To validate the quantification method, seven standards for each substrate were prepared to plot a valid calibration curve.

### Fatty acid extraction and derivatization to methyl esters

Fatty acids were extracted and converted into respective methyl esters for qualitative and quantitative analysis using a Gas Chromatography/Mass spectrometry (GC/MS) single quadrupole (QP2020 NX from Shimadzu, Kyoto, Japan). Initially, 20 mg of freeze-dried biomass were mechanically broken with the addition of 300 µL of glass beads (425–600 μm, acid washed with 0.1 M HCl), 500 µL of hexane, and 100 µL of deuterated myristic acid (d27-C14:0) as internal standard (0.98 mg/mL). The extraction was carried out using Fast Prep six cycles at 8000 rpm for 30 s, with 2-minute pause intervals. Subsequently, the tubes were centrifuged at 2000 rpm for 5 min, and the hexane phase was recovered and transferred into new tubes.

The trans-esterification and methylation protocol was carried out according to a previous study [31] with some minor changes as described below. After addition of hexane and methanol-BF_3_ to the previously extracted TAGs, the trans-esterification was conducted in a microwave digestion system (Milestone Start D, Sorisole Bergamo, Italy) set to 850 W to process 19–22 vessels simultaneously. The heating gradient started from room temperature (RT) to 120 °C within 11 min. After water addition and phase separation, the entire upper hexane phase was transferred into GC-vials, dried under N_2_ flow, and resuspended in 1 mL of hexane. The samples were then diluted and analyzed by GC/MS.

### GC/MS method for fatty acid determination

The OCFA methyl ester standards were purchased from Larodan (Solna, Sweden), while the ECFA methyl ester standards were purchased by Nu-Check Prep Inc (Minnesota, USA) (Table 1). The internal standard, deuterated myristic acid (d27-C14:0), was purchased from Merck (366889) (Darmstadt, Germany) and its methylated form was obtained from Nordic Biosite (154-28594-50) (Täby, Sweden). Concentrated stocks of all standards were meticulously prepared in hexane and stored at -20 °C in solvent-safe tubes. The separation of FAMEs was performed on Zebron (ZB-FAME) GC column (20 m L×0.18 mm ID×0.15 μm df) from Phenomenex (California, USA). The samples and standards were injected in split injection mode (1 µL at 250 °C) at split 5, and helium was used as carrier gas (1 mL/min). The method featured a temperature ramp of 25.58 min where column temperature was initially set at 50 °C hold for 2 min and then raised by proportional gradient up to 160 °C (10 °C/min), followed by a further increase to 185 °C (3 °C/min). Finally, the temperature was increased to 260 °C (20 °C/min) and held for 0.5 min. The mass transfer line and ion source were set at 250 °C. The FAMEs were ionized with Electron Impact (EI, 70 eV) and detected in scan mode (50–650 m/z). The extra peaks identified outside our available standards were qualitatively analyzed using the NIST and AMDIS spectra libraries and quantitatively assessed with the standard most closely matching their chemical structure. Samples from PA medium were run in quadruplicates, except for *R. toruloides* that exhibited poor growth but produced sufficient biomass for analyzing its fatty acids profile in triplicates. The samples from glucose medium were run in triplicates. Means and standard deviations were calculated for all the samples collected from two biological replicates.

**Table 1:** List of fatty acids analyzed in this study

IUPAC name	C-x	Common name
OCFAs*		
Heptanoic acid	C7:0	Enanthic acid
Nonanoic acid	C9:0	Pelargonic acid
Undecanoic acid	C11:0	Undecylic acid
Tridecanoic acid	C13:0	Tridecylic acid
Pentadecanoic acid	C15:0	Pentadecylic acid
(Z)-pentadec-10-enoic acid	C15:1n-5	cis-10-Pentadecenoic acid
Heptadecanoic acid	C17:0	Margaric acid
(Z)-heptadec-10-enoic acid	C17:1n-7	cis-10-Heptadecenoic acid
Nonadecanoic acid	C19:0	Nonadecylic acid
Tricosanoic acid**	C23:0	Tricosylic acid
ECFAs**		
Hexanoic acid	C6:0	Caproic acid
Octanoic acid	C8:0	Caprylic acid
Decanoic acid	C10:0	Capric acid
Dodecanoic acid	C12:0	Lauric acid
(Z)-dodec-11-enoic acid	C12:1n-1	11-Lauroleic acid
Tetradecanoic acid	C14:0	Myristic acid
(Z)-tetradec-9-enoic acid	C14:1n-5	Myristoleic acid
Hexadecanoic acid	C16:0	Palmitic acid
(Z)-hexadec-9-enoic acid	C16:1n-7	Palmitoleic acid
Octadecanoic acid	C18:0	Stearic acid
(Z)-octadec-9-enoic acid	C18:1n-9	Oleic acid
(9Z,11Z)-octadeca-9,11-dienoic acid	C18:2n-7,9	Linoleic acid
(9Z,12Z,15Z)-octadeca-9,12,15-trienoic acid	C18:3 n-3,6,9	α-Linolenic acid
Icosanoic acid	C20:0	Arachidic acid
(Z)-icos-11-enoic acid	C20:1n-9	Gondoic acid
(11Z,14Z)-icosa-11,14-dienoic acid	C20:2n-6,9	Eicosadienoic Acid
(11Z,14Z,17Z)-icosa-11,14,17-trienoic acid	C20:3n-3,6,9	Dihomolinolenic acid
(5Z,8Z,11Z,14Z)-icosa-5,8,11,14-tetraenoic acid	C20:4n-6,9,12,15	Arachidonic acid
(8Z,11Z,14Z)-icosa-8,11,14-trienoic acid	C20:3n-6,9,12	γ-Homolinolenic acid
Docosanoic acid	C22:0	Behenic acid
(Z)-docos-13-enoic acid	C22:1n-9	Erucic acid
Tetracosanoic acid	C24:0	Lignoceric acid
(4Z,7Z,10Z,13Z,16Z,19Z)-docosa-4,7,10,13,16,19-hexaenoic acid	C22:6n-3,6,9,12,15,18	Cervonic acid
(Z)-tetracos-15-enoic acid	C24:1n-9	Nervonic acid

* Methyl ester version provided by Larodan

** Methyl ester version provided by Nu-check Prep Inc

## Results

### Yeast collection

We first conducted a literature review on OCFA-producing yeasts, to guide the selection of species for this study. The final selection, comprising a total of 19 strains, was based on the following criteria: *(i)* documented capacity to produce OCFAs in sugar-based media, *(ii)* capacity to grow on and produce OCFAs from PA or combinations of different SCFAs (synthetic mixes or real SCFAs effluents), and *(iii)* oleaginous yeast species that had not been tested before for criteria *(i)* or *(ii)* (Table 2). Five strains (*L. tetrasporus*, *W. anomalus*, *Z. florentina*, *B. adeninivorans*, *R. mucilaginosa*) were selected due to reports on traces of OCFAs in their fatty acid profiles upon growth in sugar-based media. Five strains (*Y. lipolytica*, *T. delbrueckii*, *R. toruloides*, *C. cutaneum*, *C. oleaginosus*) because they had previously been cultivated in PA or SCFAs synthetic mixtures or effluents for production of OCFAs. Finally, the remaining nine strains were chosen because of their status as oleaginous yeast (reviewed in [32]), and to ensure representation of a diverse array of families within both the *Ascomycota* and *Basidiomycota* phyla. To the best of our knowledge, these latter strains have not yet been explored with respect to their abilities to produce OCFAs.

**Table 2:** List of reasons of selection of the yeast species used in this study

Taxonomy	Strain ID^1^	Reasons of selection^2^	Ref.^3^
**Ascomycota**			
Debaryomycetaceae			
*Schwanniomyces occidentalis*	Y-2470	**High TAG content**	[33]
*Debaryomyces hansenii*	Y-7426	Oleaginous yeast	[34]
Dipodascaceae			
*Yarrowia lipolytica*	W29	Can stand high [SCFAs] and [PA] g/L, prod. C17:0, C15:0	[20, 35]
		C17:1 21.3 mg/L, PA C17:1 88.6 mg/L on Ac	[13]
		C17:1 (30% total lipids), PA + G (15% total lipids) on PA	[14]
		C15:0 traces on MEA	[36]
Lipomycetaceae			
*Lipomyces tetrasporus*	Y-11,562	**Traces of C17:0 in FAs profile on YPD**	[37]
Metschnikowiaceae			
*Metschnikowia pulcherrima*	Y-7111	**Oleaginous yeast**	[38]
		Can stand up to 5 g/L PA	[39]
Phaffomycetaceae			
*Wickerhamomyces anomalus*	Y-17,698	High % of C17:1, also C15:0 and C17:0 on YPD	[27]
		**High TAG content**	[33]
*Barnettozyma californica*	Y-1680	**High TAG content**	[33]
Pichiaceae			
*Pichia occidentalis*	Y-1732	**Oleaginous yeast**	[32]r
*Saturnispora silvae*	Y-6725	Oleaginous yeast	[40]
Saccharomycetaceae			
*Torulaspora delbrueckii*	Y-2229	**C17:1 23.4 mg/L on PA**	[13]
		High TAG content	[33]
		**C17:1 (30% total lipids)**,** PA + G (18% total lipids) on PA**	[14]
*Zygotorulaspora florentina*	Y-1560	C17:1 0.49% relative FAs on YPD	[34]
Trichomonascaceae			
*Blastobotrys adeninivorans*	Y-17,692	**High % of C17:1**,** also C17:0**,** on YPD**	[27]
*Blastobotrys raffinosifermentans*	Y-27,150	Oleaginous yeast	[41]
Insertae sedis			
*Starmerella bombicola*	Y-17,069	**Oleaginous yeast**	[42]r
**Basidiomycota**			
Sporidiobolaceae			
*Rhodotorula toruloides*	CBS14	Can stand up to 5 g/L SCFAs, C17:0 3.3% relative FAs	[20]
		Can stand up to 20 g/L Ac	[43]
*Rhodotorula mucilaginosa*	Y-1593	C17:0 traces on MEA	[36]
*Rhodosporidiobolus fluvialis*	Y-12,922	Oleaginous yeast	[44]
Trichosporonaceae			
*Cutaneotrichosporon oleaginosus*	ATCC 20,509	SCFAs was shown to be a favorable feedstock	[20, 45]
		**45.1% OCFAs on SCFAs mix**	[21]
*Cutaneotrichosporon cutaneum*	Y-2526	C17:1 67 mg/L on PA	[13]
		C17:1 (28% total lipids), PA + G (12% total lipids) on PA	[14]

^1^ Strains used in the present study

^2^ Bold text is used to highlight cases where the same strain was used in this study as in the referenced one

^3^ r behind the reference number indicates that the reference is a review

SCFAs, short chain fatty acids; Ac, acetic acid; PA, propionic acid; G, glucose; FAs, fatty acids; TAGs, triacylglycerols; MEA, malt extract agar; YPD, Yeast extract, Peptone, D-glucose

### Growth profiles in glucose based medium

Initially, we assessed the growth capabilities of all strains in glucose minimal medium at molar C/N ratio 9 and 50 in 96-well microtiter plates and determined the maximum specific growth rates and the duration of the lag phases.

**Fig. 2:**
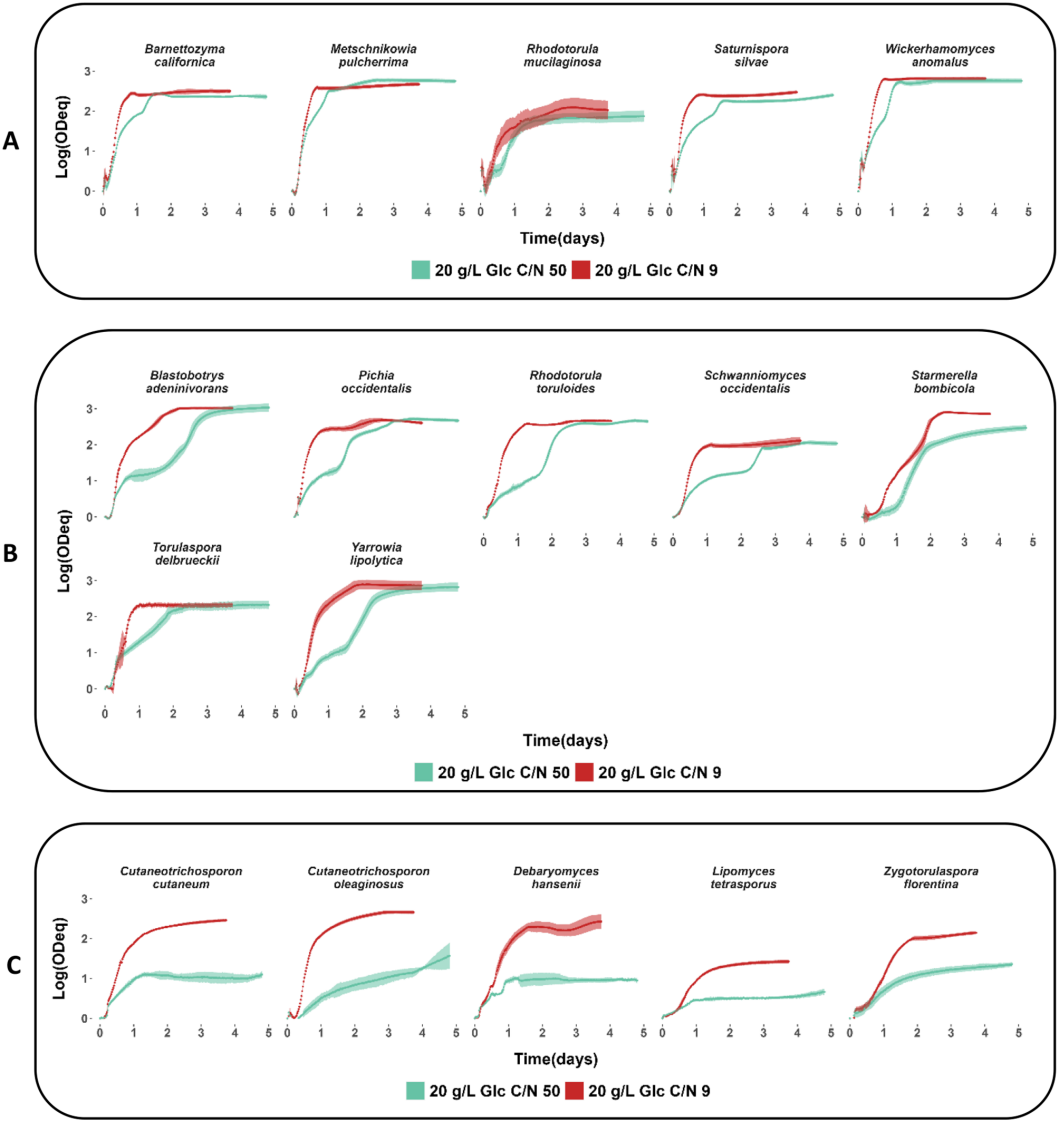
Comparison of growth profiles in glucose-based media with high and low C/N ratios. Yeast strain growth was analyzed in 20 g/L glucose at molar C/N ratios of 9 and 50. The graphs display data obtained from a Growth Profiler in a 96-well plate system. Data are shown as mean ± standard deviation for biological triplicates. The y-axis shows the biomass formed during cultivation on a logarithmic scale using *green values*, considered equivalent to OD, plotted against time (days) on the x-axis. **A**) strains with similar growth profiles at C/N ratios of 9 and 50. **B**) strains with distinct biphasic growth at C/N ratio 50, reaching the same or almost the same final ODeq as in C/N ratio 9. **C**) strains with non-biphasic growth at C/N ratio 50, lower maximum specific growth rates, and lower final ODeqs than those observed at C/N ratio 9

At a molar C/N ratio of 9, all strains exhibited short lag phases (≤ 14 h) and reached maximum specific growth rates between 0.15 and 0.30 h⁻¹ (Suppl. Figure S1). By comparison, growth profiles at a C/N ratio of 50 showed clear strain differences (Fig. 2). *B. californica*, *M. pulcherrima*, *R. mucilaginosa*, *S. silvae* and *W. anomalus* displayed similar growth profiles in C/N ratio 50 as in C/N ratio 9, but with accentuated deceleration phases, reaching the same or almost the same final ODeq as in C/N ratio 9 only a few hours later. In contrast, strains such as *B. adeninivorans*, *P. occidentalis*, *R. toruloides*, *S. occidentalis*, *S. bombicola*,* T. delbrueckii* and *Y. lipolytica* displayed growth profiles in C/N ratio 50 that were clearly different from those in C/N ratio 9, with pronounced biphasic growth, lower maximum specific growth rates and longer time needed to reach the same final ODeq as in C/N ratio 9. *C. cutaneum*,* C. oleaginosus*,* D. hansenii*,* L. tetrasporus*, and *Z. florentina* primarily exhibited non-biphasic growth profiles, with low maximum specific growth rates (0.02–0.09 h⁻¹). These strains also failed to achieve the final ODeq observed under C/N ratio of 9 within the 5-days experimental timeframe. We can conclude that the growth characteristics were clearly influenced by the C/N ratio in a species-dependent manner.

### Fatty acids profiles resulting after cultivation in glucose-based medium

Next, we determined the yeasts’ capacities to produce fatty acids in minimal medium containing 20 g/L glucose at molar C/N ratio 50. At the end of cultivation (115 h), when all the strains had reached stationary phase, we sampled and determined cell dry weight (g/L), residual glucose (g/L) and fatty acid profiles. Unfortunately, *C. cutaneum*, *D. hansenii*, *L. tetrasporus*, and *Z. florentina* did not form enough biomass in glucose-based medium to enable all measurements (including technical replicates for fatty acid profile analysis). Consequently, the analyses were performed on the 15 yeasts that produced high enough cell mass concentrations.

**Fig. 3:**
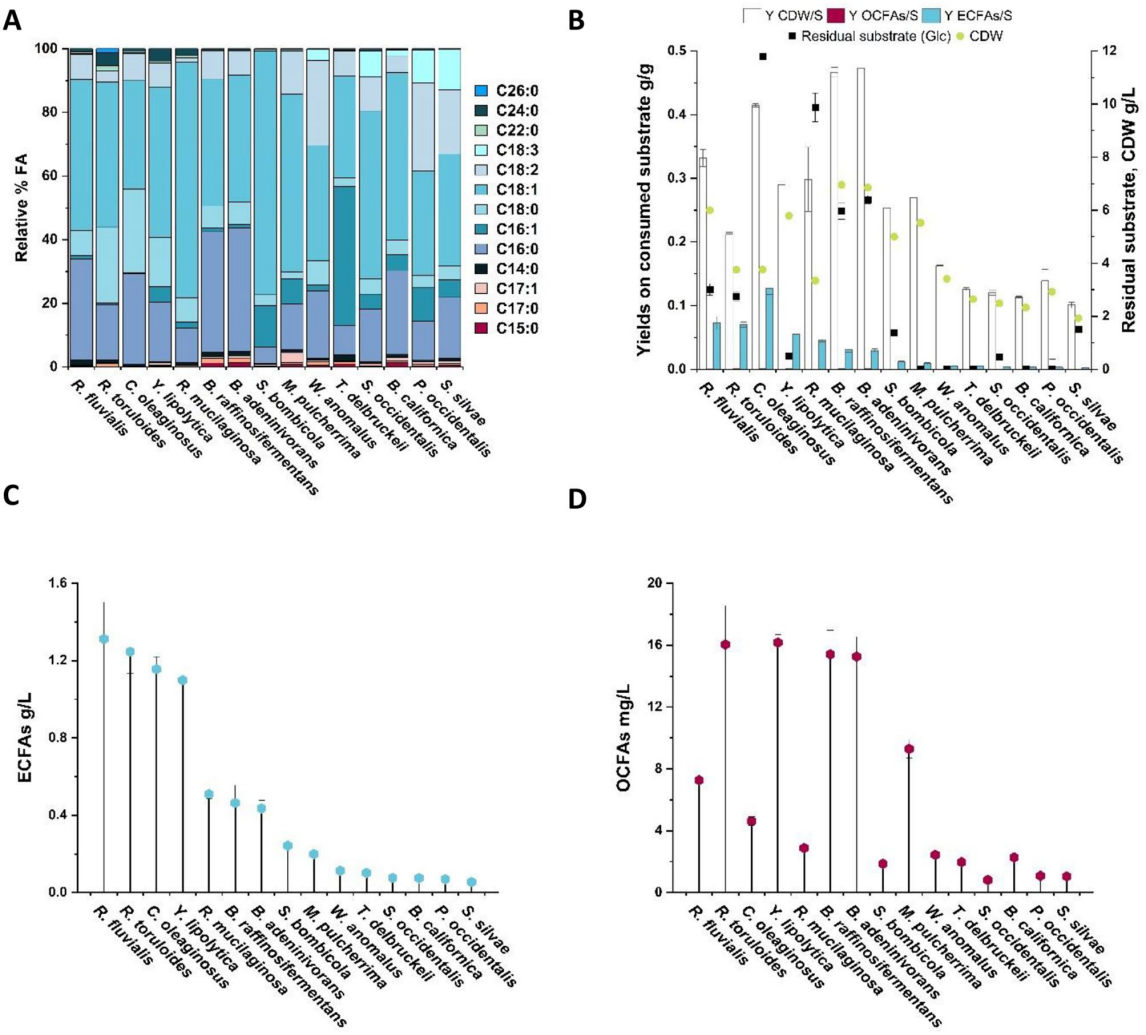
Results from fatty acids analysis represented with different metrics. The 15 strains that produced high enough cell mass titers when cultivated in 20 g/L glucose at molar C/N ratio 50 are displayed. All plotted data are ordered by decreasing ECFAs g/L metric. **A**) Relative percentages ratio (%) of single fatty acids on total fatty acids profile; **B**) Histograms representation of yields on consumed substrate (S) in g/g of final biomass (CDW/S), final odd chain fatty acids (OCFAs/S) and final even chain fatty acids (ECFAs/S). Overlapping scatter point of final residual substrate (g/L) and final CDW (g/L) relative to each strain; **C**) titer in g/L of the final total amount of ECFAs; **D**) titer in mg/L of the final total amount of OCFAs

Analysis revealed that ECFAs are the predominant fatty acids produced under glucose-based conditions, accounting for 95.0-99.5% of the total fatty acids across all studied strains (Fig. 3A). The percentage of specific fatty acids on total fatty acids differed between the different yeasts, although palmitic acid (C16:0), palmitoleic acid (C16:1), stearic acid (C18:0), oleic acid (C18:1) andlinoleic acid (C18:2) were among the most abundant fatty acids for all strains. *S. occidentalis*, *P. occidentalis*, *S. silvae*, *W. anomalus* and *B. californica* produced also α-linolenic acid (C18:3), with levels ranging between 1.9% and 12.7%. Most yeasts produced more C16:0 than C16:1, except in *T. delbrueckii* and *S. bombicola* where C16:1 was more abundant than C16:0. On the contrary, the unsaturated fatty acids C18:1 and C18:2 were generally the preferred ones compared to saturated C18:0. Very long chain fatty acids (VLCFAs) such as behenic acid (C22:0) were produced by *R. toruloides* (1.3%), and lignoceric acid (C24:0) was mainly produced by *R. fluvialis*, *R. toruloides*,* R. mucilaginosa* and *Y. lipolytica* (1.0, 4.1, 2.2 and 3.9% respectively). Notably, *R. toruloides* also produced a small amount (1.2%) of cerotic acid (C26:0), highlighting the capability of this yeast to produce VLCFAs [46].

In terms of OCFAs, pentadecanoic acid (C15:0), heptadecanoic acid (C17:0) and heptadecanoic acid (C17:1) were the main fatty acids produced. *M. pulcherrima* produced the highest percentage of OCFAs, almost 5% of the total fatty acids, followed by the two *Blastobotrys* strains *B. raffinosifermentans* and *B. adeninivorans* (3.4%), which exhibited very similar fatty acid profiles overall, and *B. californica* (3.1%). Whereas all strains displayed relatively low OCFA yields, the yields of ECFAs and yeast cell biomass on consumed substrate varied substantially between strains (Fig. 3B). *C. oleaginosus* exhibited the highest ECFAs yield on consumed substrate (0.13 g/g), followed by *R. fluvialis*, *R. toruloides*, *Y. lipolytica* and *R. mucilaginosa* (0.07, 0.07, 0.06, 0.05 g/g, respectively). *B. raffinosifermentans* and *B. adeninivorans* on the other hand exhibited the highest yeast biomass titers (6.95 and 6.85 g/L, respectively) and a CDW yield of 0.47 g/g, but lower ECFAs yields (0.03 g/g for both). This suggests that these yeasts prioritized carbon allocation toward biomass production rather than lipid synthesis under the tested condition. All other analyzed strains reached ECFAs yields of less than 0.02 g/g.

Overall, the titers indicated that *R. fluvialis*, and *R. toruloides*, *C. oleaginosus* and *Y. lipolytica* were amongst the best fatty acid producers (1.09–1.31 g/L ECFAs) (Fig. 3C). In contrast, *R. mucilaginosa*, *B. raffinosifermentans*, and *B. adeninivorans* produced an intermediate amount of ECFAs (0.43 to 0.50 g/L), while the remaining yeast strains showed a poor production of ECFAs (0.05 to 0.24 g/L). The OCFA titers for all strains were negligible, although some differences were observed, within the range of 1–16 mg/L, and where the top four OCFA-producing yeasts were *R. toruloides*,* Y. lipolytica*,* B. raffinosifermentans*, and *B. adeninivorans* (Fig. 3D).

### Growth in increasing concentrations of propionic acid

We assessed the growth capabilities of all 19 yeast strains in minimal medium with six different concentrations of PA (g/L): 5, 10, 15, 19, 24, and 29, and buffered to pH 6.8. Only those strains that displayed growth at lower concentrations of PA were included in the trials with higher concentrations. It is important to note that PA has dual roles, functioning both as both a carbon and energy source while also acting as a weak acid that imposes cellular stress. This complexity makes interpreting growth patterns more challenging. With increasing PA concentrations, cells have access to more carbon for biomass formation, but the accompanying stress can lead to longer lag phases, reduced specific growth rates, and/or lower final optical density (ODeq).

**Fig. 4:**
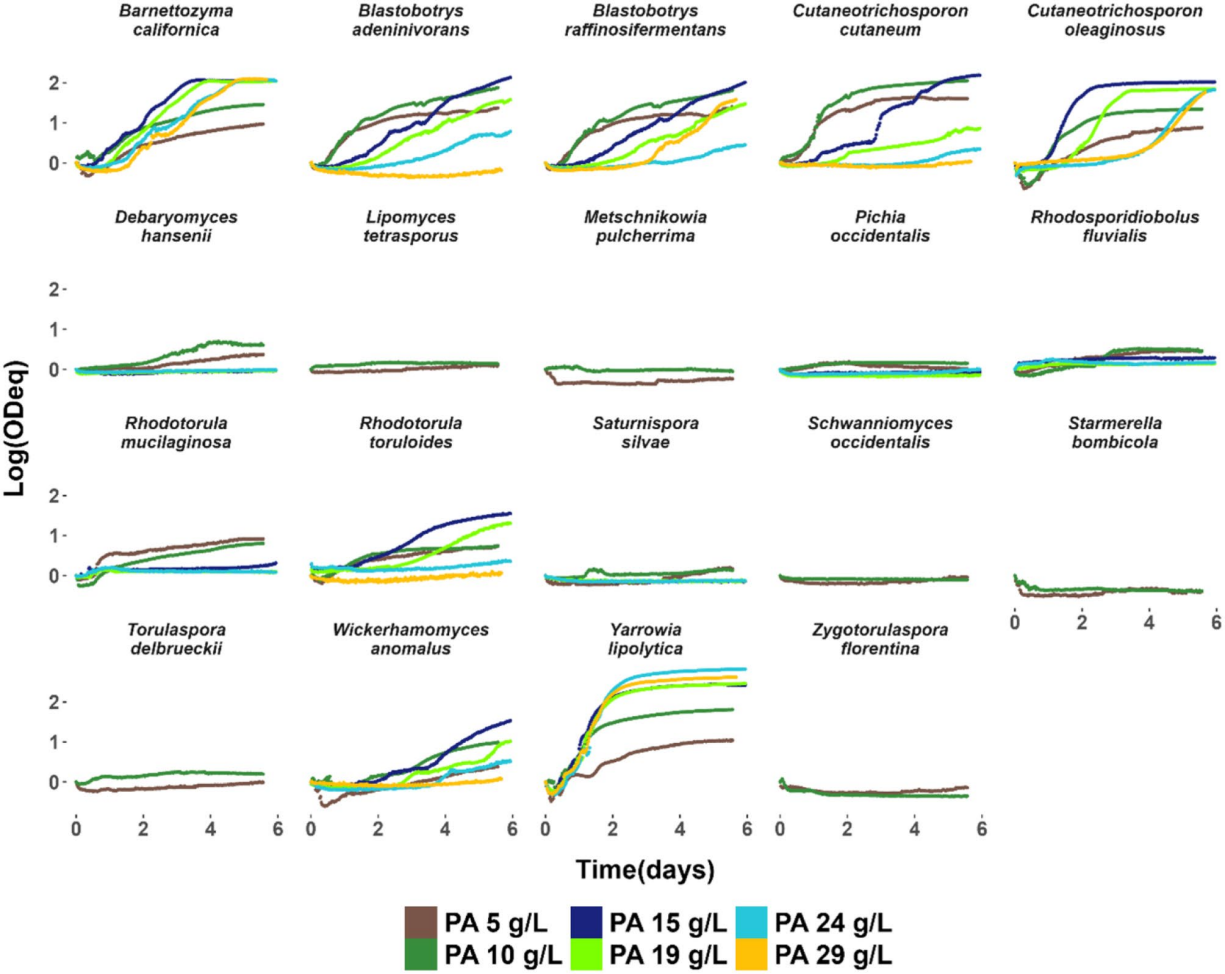
Comparison of growth profiles in increasing concentrations of propionic acid. Yeast strain tolerance to PA were analyzed in 5, 10, 15, 19, 24, and 29 g/L of PA at molar C/N ratio of 9 following the growth using a Growth Profiler in a 96-well plate system. Data are shown as means of three biological replicates. The y-axis shows the biomass formed during cultivation on a logarithmic scale using *green values*, considered equivalent to OD, plotted against time (days) on the x-axis

Eight strains could clearly grow on PA concentrations up to 24 g/L: *B. adeninivorans*, *B. raffinosifermentans*, *B. californica*, *C. cutaneum*, *C. oleaginosus*, *R. toruloides*, *W. anomalus* and *Y. lipolytica* (Fig. 4). At 24 g/L and 29 g/L of PA, *Y. lipolytica*, *B. californica*, and *C. oleaginosus* exhibited higher specific growth rates than the other strains. *Y. lipolytica* displayed the most robust growth characteristics on PA with short lag phases and high final ODeq, while *B. californica* and *C. oleaginosus* displayed extended lag phases, although they eventually reached the same final ODeq as in 19 g/L PA. The other five yeasts showed the highest ODeq in 15 g/L PA, though some strains did not appear to reach stationary phase within the experimental timeframe.

Conversely, all 19 strains grew when PA (5–10 g/L) was supplemented with 2 g/L of glucose (Suppl. Fig. S2). However, *L. tetrasporus*, *M. pulcherrima*, *S. occidentalis*, *S. bombicola*, *W. anomalus*, and *Z. florentina* did not reach the same final ODeq as the control with 2 g/L of glucose alone. This suggests that these strains experience greater cellular stress in response to PA compared to the other strains.

### Fatty acids profiles in propionic acid-based medium

The eight strains that could grow on PA at C/N ratio 9 were next cultivated in 15 g/L of PA and at C/N ratio 50 to promote fatty acids accumulation. All eight strains displayed growth profiles similar to those in C/N ratio 9 (Suppl. Fig. S3), in contrast to the varying profiles observed for most strains in glucose-based cultivation at C/N ratios 9 and 50 (Fig. 1). The microtiter growth cultivations revealed that 188 h was an appropriate harvesting time, as all strains had ceased to grow at this time (Suppl. Fig. S3). We cultivated the eight PA tolerant yeasts in shake flasks with medium containing 15 g/L of PA at molar C/N ratio 50, and samples were collected and used to determine the cell dry weight (g/L), the residual substrate (g/L) and fatty acid profile. Unfortunately, *W. anomalus* did not grow to high enough cell mass titers in shake flasks, despite its evident growth in the microtiter plate, and was therefore left behind.

**Fig. 5:**
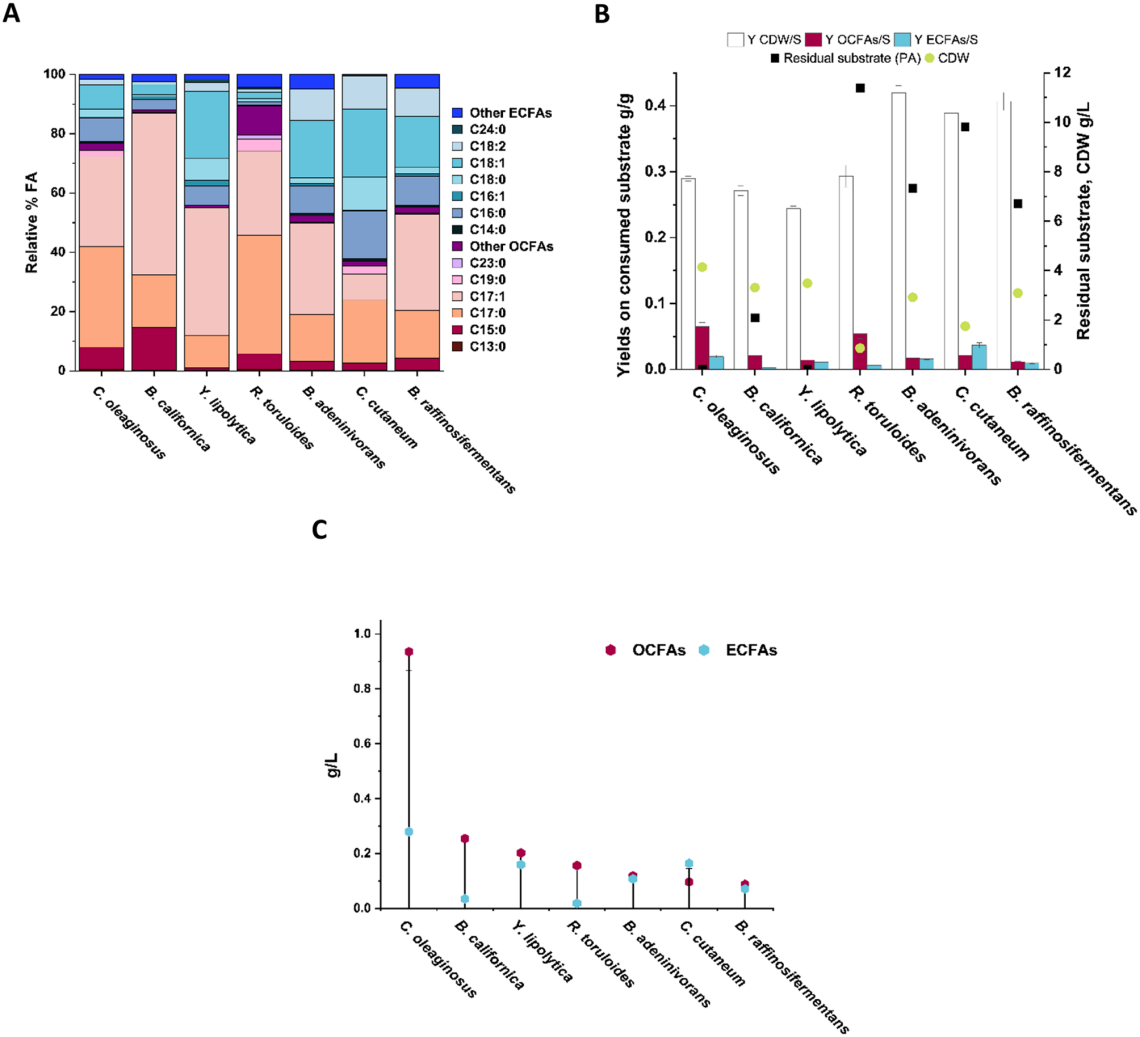
Fatty acids profiles of seven strains cultivated on 15 g/L propionic acid at molar C/N ratio 50. **A**) relative % fatty acid profiles. Other ECFAs: 7-C16:1, 9,12-C18:2. Other OCFAs: 10-C19:1, C21:0, C25:0; **B**) yields on substrate (S) of biomass (CDW/S), odd chain fatty acids (OCFAs/S) and even chain fatty acids (ECFAs/S), overlapping illustrations of residual substrate (g/L) and CDW (g/L) to show the full potential of every analyzed yeast; **C**) titer (g/L) of the total amount of odd chain and even chain fatty acids

All seven yeasts showed fatty acid profiles rich in OCFAs, but the ratio of OCFAs on total fatty acids varied significantly (Fig. 5A). *R. torulodies* and *B. californica* produced close to 90% of their total fatty acids as OCFAs, while *C. oleaginosus* produced about 80%. *Y. lipolytica* and the two *Blastobotrys* strains produced 55% OCFAs, and *C. cutaneum* 37%. Moreover, the percentage of individual fatty acids on total fatty acids varied between yeasts, although all accumulated preferentially C15:0, C17:0 and C17:1 (similar to the OCFA-profiles when using glucose as substrate). *R. toruloides*, *C. oleaginosus* and *C. cutaneum* also produced measurable amounts of nonadecanoic acid (C19:0), and *R. toruloides* generated 1.44% of its total fatty acids as tricosilyc acid (C23:0). The primary ECFAs accumulated were C16:0, C18:0, C18:1 and C18:2.

We also identified additional OCFAs and ECFAs peaks, not present in our standards. These are assigned as “other OCFAs” or “other ECFAs” in Fig. 5A. All yeast species except *Y. lipolytica* produced nonadecenoic acid (10-C19:1). *R. toruloides* also produced a substantial relative amount of heneicosanoic acid (C21:0, 1.86%) and pentacosylic acid (C25:0, 6.83%) (Fig. 5A) which, together with C23:0, underline the capability of this yeast to produce VLCFAs also on PA. We also detected the “other ECFAs” hexadecenoic acid (7-C16:1), octadecadienoic acid (9,12-C18:2) in all strains except *C. cutaneum*.

*C. oleaginosus* and *R. toruloides* displayed the highest OCFA yields on consumed PA (0.07 and 0.05 g/g respectively), while the other five yeasts displayed more modest yields (0.01–0.02 g/g) (Fig. 5B). Moreover, *C. oleaginosus*,* Y. lipolytica* and *B. californica* used most of the available carbons, whereas the other strains used substantially less (2.9–7.6 g/L of the initial 15 g/L). *C. oleaginosus* and *C. cutaneum* exhibited the highest ECFA yields based on consumed substrate (0.02 and 0.04 g/g respectively). The three strains that demonstrated the most efficient PA conversion also reached the highest fatty acid titers. *C. oleaginosus* produced 1.22 g/L of total fatty acids, with OCFAs accounting for 0.94 g/L. *B. californica* reached 0.29 g/L of total fatty acids, of which 0.26 g/L were OCFAs, and *Y. lipolytica* produced 0.36 g/L of total fatty acids, with 0.20 g/L as OCFAs (Fig. 5C).

In conclusion, all the yeast species cultivated in 15 g/L of PA showed profiles enriched in OCFAs compared to their fatty acid profiles from glucose-based cultivation, although there were clear differences between strains in terms of PA uptake and conversion and the accumulated fatty acid profiles.

### Comparison of fatty acid profiles in glucose and propionic acid

Finally, we compared the yeasts’ capacities to produce fatty acids on glucose and PA by evaluating the percentage of fatty acids relative to dry yeast biomass, as well as the fatty acid profiles and abundance of individual fatty acids produced.

**Fig. 6:**
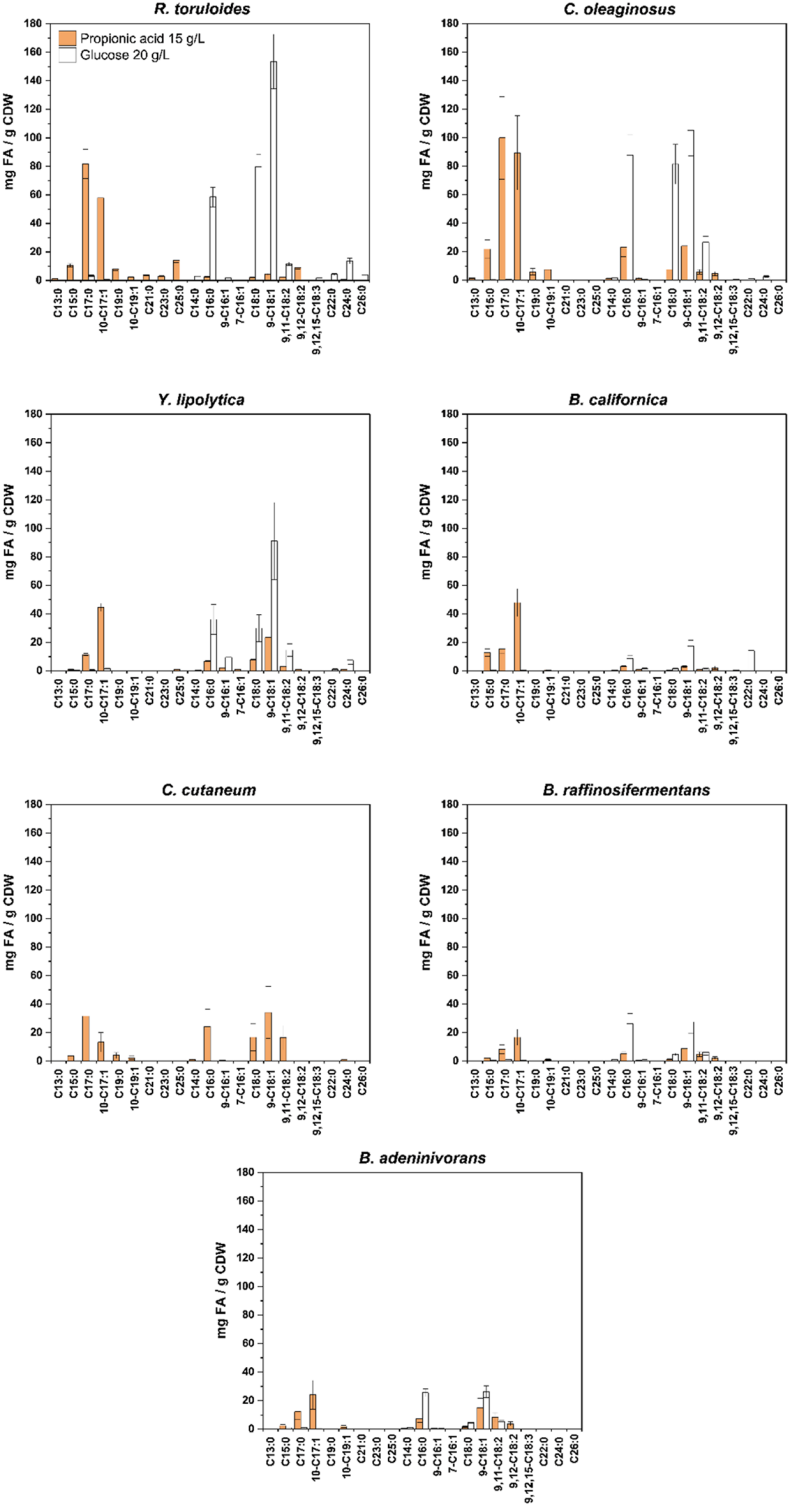
Comparison between the fatty acid profiles in 15 g/L of PA and 20 g/L of glucose at molar C/N ratio 50. The y-axis shows the µg fatty acids produced per mg of biomass (CDW). The x-axis shows the labels of the single fatty acid present in the profile. The orange and white bars represent the PA and glucose conditions, respectively

In PA-based medium, all yeast strains exhibit a more diverse fatty acid profile compared to those in glucose-based medium, as indicated by the presence of a mix of both OCFAs and ECFAs (Fig. 6). Interestingly, *C. cutaneum* demonstrated poor growth in glucose-based medium at a C/N ratio of 50 (unpublished data), which prevented us to proceed with downstream measurements, compared to PA-based medium, where we obtained 1.75 g/L of CDW (Fig. 5A), indicating a potential preference for PA as a substrate. In glucose-based medium, *R. toruloides*, *C. oleaginosus* and *Y. lipolytica* showed high levels of C16:0, C18:0 and C18:1, while the other strains contained mainly C16:0 and C18:1. In PA-based medium, all strains exhibited strain-dependent production of ECFAs, yet all continued to produce substantial amounts of C16:0 and C18:1. The consistency in the production of these ECFAs in presence of different carbon sources likely reflect the importance of these fatty acids for membrane structures [47, 48].

*R. toruloides* and *C. oleaginosus* demonstrated superior oleaginous capabilities in the presence of both carbon sources, accumulating 33.6% (w/w) and 30.9% (w/w) of fatty acids on dry biomass in glucose, and 20.4% (w/w) and 29.4% (w/w) in PA, respectively (Table 3). Notably, *C. oleaginosus* accumulated similar amounts of total fatty acids per g of dry biomass in both conditions (Fig. 6), although it produced more total fatty acids per g of carbon consumed in glucose-based media (0.32 g/g-C) compared to PA media (0.17 g/g-C) (Table 3). *Y. lipolytica*, which demonstrated superior growth on PA (Fig. 4), produced comparatively fewer grams of fatty acids from this carbon source compared to glucose (0.05  vs. 0.14 g/g-C) (Table 3). Interestingly, *B. californica* was the only yeast that produced more total fatty acids per g of consumed carbon on PA compared to glucose-based media (0.05 vs. 0.01 g/g-C). This suggests that PA is a more favorable substrate than glucose for promoting oleaginicity in *B. californica*, as it appears to channel more carbon towards lipid accumulation under PA growth.

**Table 3:** Comparison of metrics between glucose and propionic acid-based cultivations

	% tot FAs / CDW (w/w)		g tot FAs-produced / g C-consumed	
Strain	Glucose	PA	Glucose	PA
*R. toruloides*	33.62 ± 3.08	20.39 ± 1.41	0.18 ± 0.01	0.12 ± 0.00
*C. oleaginosus*	30.86 ± 1.72	29.38 ± 2.23	0.32 ± 0.02	0.17 ± 0.01
*Y. lipolytica*	19.17 ± 0.25	10.38 ± 0.30	0.14 ± 0.00	0.05 ± 0.01
*B. californica*	3.32 ± 0.35	8.75 ± 1.56	0.01 ± 0.00	0.05 ± 0.00
*C. cutaneum*	-	14.91 ± 1.70	-	0.12 ± 0.01
*B. raffinosifermentans*	6.89 ± 1.34	5.15 ± 0.61	0.08 ± 0.01	0.04 ± 0.01
*B. adeninivorans*	6.49 ± 0.62	7.77 ± 0.04	0.08 ± 0.01	0.07 ± 0.00

The deviation from the mean is calculated between two biological replicates

FAs, fatty acids; CDW, cell dry weight; C, carbon; tot, total

## Discussion

In this study, we screened 19 yeast strains from different species for their capacity to convert glucose and PA into OCFAs and ECFAs. The selected yeast strains, primarily oleaginous species from different phylogenetic families and phyla, span a broad evolutionary range and provided a unique platform for comparative phenomics. Moreover, the limited metabolic and lipidomic data available for many of these strains [32] offered an exciting opportunity to uncover novel metabolic capabilities.

In our screening setup, we used similar growth conditions and sampling time points for all strains, allowing parallel cultivations and straight-forward comparisons. However, as the strains responded vastly differently to the various cultivation conditions - evidenced by their growth profiles and capacities to convert carbons into yeast biomass and lipids - the limited experimental space may have constrained the range of metabolic capacities observed. For example, yeasts are capable of lipid turnover, where they can consume previously accumulated storage lipids during extended cultivation. This makes the correct time point for sampling important but difficult to predict [26, 49]. Additionally, we observed that for certain strains, including *B. californica*, *M. pulcherrima* and *W. anomalus*, there were no significant differences in the growth curves on glucose-based medium at molar C/N ratios of 9 and 50. This suggests that a C/N ratio of 50 may not have imposed nitrogen limitation for these strains, which likely affected the lipid accumulation negatively. Under glucose-based cultivation conditions, we observed only minimal production of OCFAs, more specifically 0.5-5% of the total fatty acids. This result agrees well with most reported studies so far [34, 36], although it should be noted that OCFAs are often excluded from the fatty acid analysis using GC/MS of glucose-grown cells, and thus there is a lack of information about potential OCFA production for many strains. Among the yeasts that we assessed, *M. pulcherrima* produced the highest proportion of OCFAs (5% of total fatty acids) when grown on glucose, although the overall OCFA yield and titer remained relatively low. *B. adeninivorans* and *W. anomalus* produced more OCFAs than average (3.4% and 2.1% of total fatty acids, respectively), but far from the 30% reported by Olstorpe and colleagues [27]. In their study, the authors used rich yeast medium (YPD), whereas we instead used minimal medium with glucose and a molar C/N ratio of 50 to induce nitrogen starvation. The discrepancies in experimental conditions and outcomes indicate that optimized conditions can substantially elevate the OCFA/ECFA ratio. In terms of total fatty acids on glucose, the well-studied yeasts *R*. *toruloides*, *C. oleaginosus* and *Y. lipolytica* and the relatively under-explored yeast *R. fluvialis* stood out as the best performing strains, both in terms of yields and titers. Many of the strains that accumulated medium-to-large amounts of lipids also exhibited higher CDWs compared to strains with low fatty acid accumulation. This could at least in part be attributed to the fact that intracellular lipids contribute to the overall CDW.

In contrast to the well-studied glucose catabolism, which is believed to be highly conserved between yeast species [50], there is still very little knowledge on how PA is taken up and metabolized in yeasts. Furthermore, there is little known about the mechanisms that trigger fatty acid production and accumulation during growth on PA. Besides being a potential carbon source, PA is a weak acid that poses stress to the cells in a pH dependent manner. At pHs lower than the pKa of PA (4.87), the undissociated form of PA predominates, which facilitates rapid diffusion across the plasma membrane [51]. Once inside the cell, PA dissociates and acidifies the cytoplasm, disrupting metabolic processes and consuming cellular energy to mitigate the acid stress [51, 52]. In line with this, strains displayed very poor or no growth on PA (5 and 10 g/L) at pH 5.0 (unpublished results). To mitigate the acid stress, we cultivated the yeasts in phosphate-buffered media at pH 6.8, where most of the acid is in its dissociated form, allowing slower and more controlled import and reducing cellular stress, as shown for acetic acid [53, 54]. Despite this adjustment, 11 out of 19 strains were still unable to grow on PA. This result correlates well with the findings in a recent publication, where only a small subset of the > 1400 yeast strains tested could grow on mixes of SCFAs (including PA) [55]. The eight strains in our collection that could grow on 15 g/L of PA as the sole carbon source are therefore interesting for further exploration, as they possess metabolic routes to convert propionate into biomass and energy as well as into lipids.

For comparative reasons, we used C/N ratio 50 both for PA- and glucose-based fatty acid production. During PA-based cultivations, many of the growth profiles in C/N ratios 9 and 50 were similar, suggesting that the molar C/N ratio of 50 may not have imposed nitrogen limitation. In fact, previous studies have reported that C/N ratios as high as 200 in SCFA/PA-based media can optimize fatty acid yields, albeit at the expense of yeast biomass formation and fatty acid titers [43, 56]. Thus, despite observing fatty acid titers and yields comparable to those seen in glucose cultivations at a C/N ratio of 50, there may be potential for further optimization of OCFA yields by fine-tuning the C/N ratio in a strain-specific manner.

We observed substantial variations in both fatty acid titers, yields and profiles across yeast strains grown on PA, with OCFAs comprising 37–89% of total fatty acids. *C. oleaginosus* stood out as the top OCFAs producer in our dataset, both in terms of titers (0.94 g/L) and yields (0.07 g/g). Notably, it consumed all PA available within 188 h and produced about 80% of OCFAs of total fatty acids on PA, mainly C17:0 and C17:1 (in equal amounts). These results are in strong agreement with findings reported by Liu and colleagues, who cultivated *C. oleaginosus* under conditions close to those in our study, with the primary difference being their use of a C/N ratio of 100 [21]. In contrast, our findings differ from those of Woo Park et al. [57], who cultured the yeast at pH 5.5 and used NaNO₃ as the nitrogen source, highlighting the significant impact of pH and nitrogen source on yeast performance in PA. Moreover, strain performance and lipid accumulation can be enhanced through metabolic engineering, as genetic tools have been developed for *C. oleaginosus*, although the high GC content of the genomic DNA in this yeast can be a limiting factor for straight forward genetic manipulations [58, 59].

*Y. lipolytica* displayed the shortest lag phases and highest final ODeqs during growth on PA concentrations 24 and 29 g/L among the strains tested. This is consistent with literature, where this yeast species has been reported to grow in pure PA or mixtures of SCFAs at concentrations of $\:\ge\:$15 g/L [17, 20, 35]. Gao et al. in [35] demonstrated that the *Y. lipolytica* strain CICC 31,596 could achieve a remarkable titer of 4.5 g/L fatty acids and a CDW of 18 g/L on 50 g/L PA as the sole carbon source, with an initial pH of 8 and a molar C/N ratio of 100 employing NH_4_Cl as the N-source. In our study, *Y. lipolytica* consumed all available PA (15 g/L), but in comparison to *C. oleaginosus*, it converted less of the consumed carbon into fatty acids (0.01 g/g, 0.36 g/L of total fatty acids), and produced 55% OCFAs of total fatty acids, preferentially C17:1. With OCFA production in *Y. lipolytica* being relatively well-studied, combined with its advanced genetic tools and genome-scale metabolic models [60, 61], this yeast emerges as a highly promising host for OCFA production.

*R. toruloides* exhibited the highest OCFAs ratio of the strains assessed, with OCFAs constituting 90% of its total fatty acids. Consistent with its well-documented production of long-chain ECFAs under glucose-based conditions [46], it also produced the longest OCFAs among all the yeasts evaluated (C25:0). While its OCFA yield on consumed PA was comparable with that of *C. oleaginosus*. Nevertheless, *R. toruloides* struggled to grow on and metabolize PA effectively, resulting in total fatty acid titers of only 0.18 g/L. Similarly, Llamas et al. [20] observed that *R. toruloides* NRRL-Y-27,012 could not grow in SCFA effluent concentrations exceeding 10 g/L, exhibiting a prolonged lag phase even at this relatively low concentration. Combined, these findings suggest that *R. toruloides’* sensitivity towards SCFAs prevents efficient proliferation and lipid accumulation on these carbon sources.

## Conclusions

This study underscores the significant potential of oleaginous yeasts for production of OCFAs but also emphasizes the substantial gaps in our understanding of OCFAs biosynthesis and the need for further fundamental and applied research. By comparing 19 distantly related strains, we observed striking differences in nitrogen starvation responses, PA catabolism, and fatty acid production profiles. A deeper physiological and metabolic understanding of key OCFA producing strains, including but not limited to *C. oleaginosus*, *Y. lipolytica*, and *R. toruloides*, would greatly facilitate process optimization that can lead to improved OCFA yields and productivities. Future work also involves elucidating the metabolic pathways and regulatory mechanisms critical for converting propionic acid into OCFAs, as well as uncovering the genetic basis of tolerance to elevated PA concentrations. Such knowledge will provide a foundation for targeted strain engineering and adaptive laboratory evolution to enhance cell factory performance. Since propionic acid is a key precursor for OCFA production and can be generated alongside other short-chain fatty acids via anaerobic fermentation, our work presents new opportunities for leveraging industrial waste streams to produce valuable microbial lipids through sustainable production processes.

## Electronic supplementary material

Below is the link to the electronic supplementary material.

Supplementary Material 1

## Data Availability

The datasets generated and analyzed in the study are available from the corresponding author on reasonable request.

## Abbreviations

OCFA: Odd chain fatty acid
ECFA: Even chain fatty acid
PA: Propionic acid
GC/MS: Gas Chromatography/Mass spectrometry
HPLC: High Pressure Liquid Chromatography
SCFA: Short chain fatty acid
CDW: Cell dry weight
S: Substrate
VLCFA: Very long chain fatty acid
