# Supplementary material for: Impact of glucose and propionic acid on even and odd chain fatty acid profiles of oleaginous yeasts
Source: BMC Microbiol. 2025 Feb 18;25:79. doi: 10.1186/s12866-025-03788-w (PMC11834278; doi:10.1186/s12866-025-03788-w)
Supplement: Supplementary file 1 — Supplementary Material 1 [file 12866_2025_3788_MOESM1_ESM.docx]

# Supplementary Information


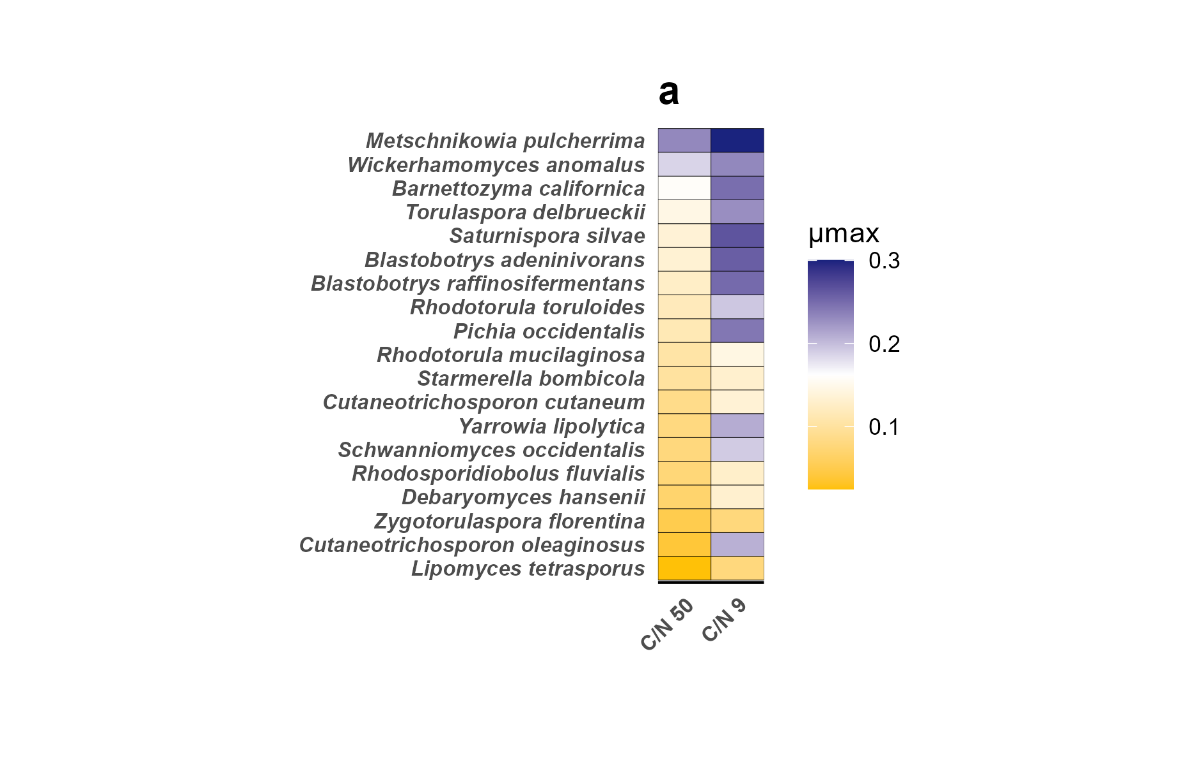

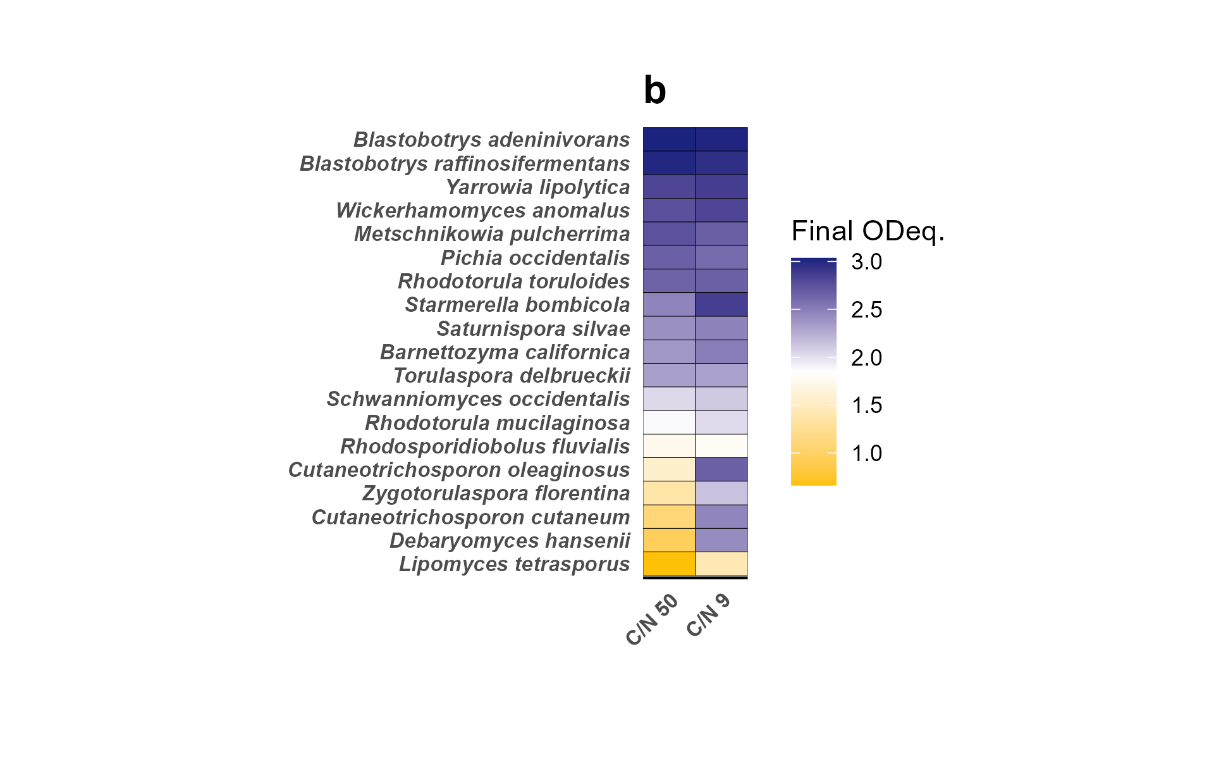


**A**

**B**

**Figure S1. Heat maps of maximum specific growth rate and final ODeq. of the strains grown in 20 g/L glucose at molar C/N ratios 9 and 50.** The graphs display maximum specific growth rate (**A**) and final ODeq reached after about 4-5 days of cultivation (**B**) by all strains grown in 20 g/L of glucose at low and high C/N ratio. The strains are sorted in descending order for the C/N ratio 50 condition. The maximum specific growth rate has been calculated in R using the *all_spline* function. This function intercepts the first inflection point of the curve using an exponential function.

**
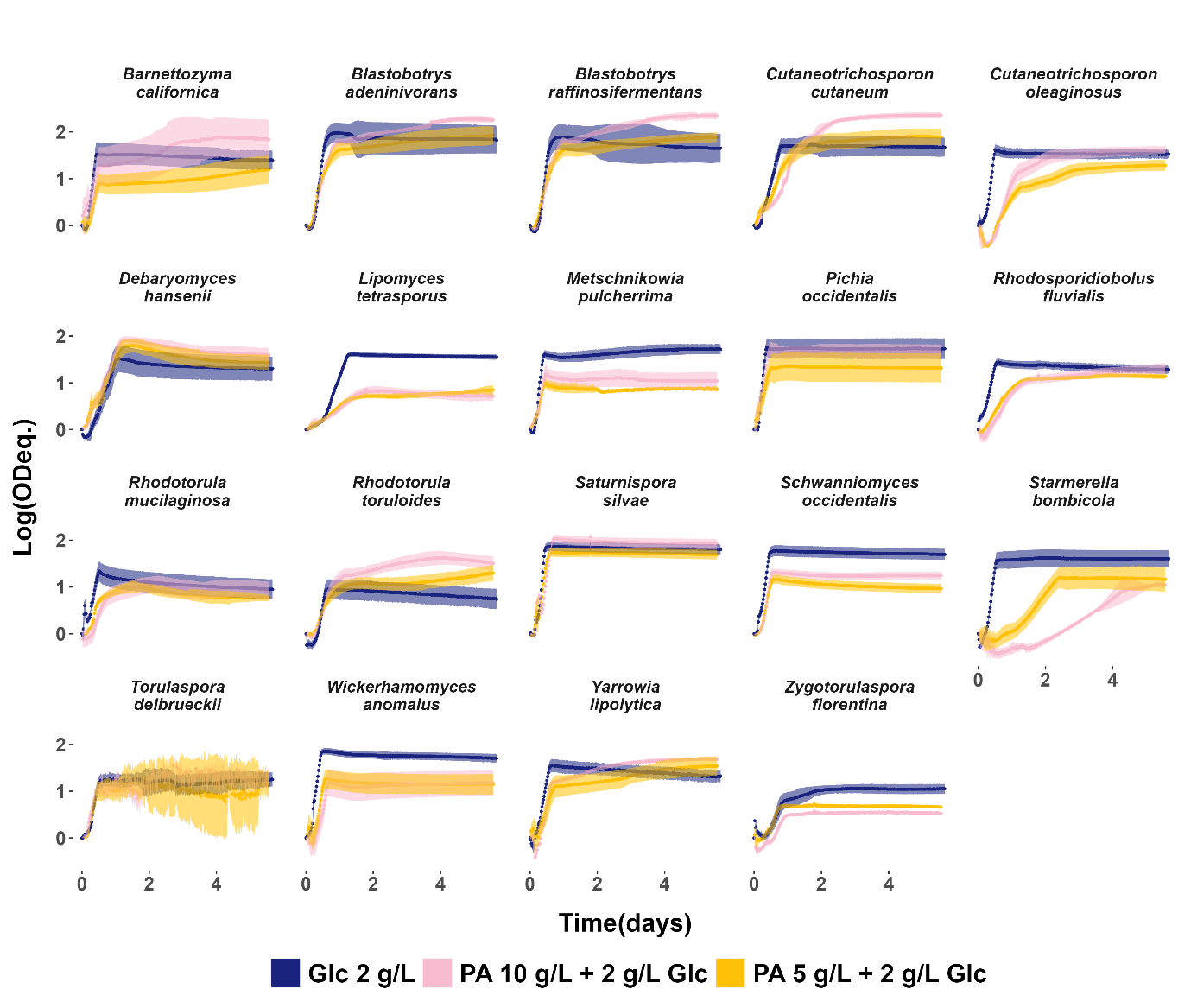
**

**Figure S2. Comparison of growth profiles in 2 g/L glucose with or without addition of 5 and 10 g/L of PA, respectively.** The graphs display data obtained from a Growth Profiler in a 96-well plate system. Data are shown as mean ± standard deviation for biological triplicates. The y-axis shows the biomass formed during cultivation on a logarithmic scale using green values, considered equivalent to OD, plotted against time (days) on the x-axis. The Growth Profiler utilizes image analysis, capturing pictures from the transparent bottom of the 96-well plate every 30 minutes to measure cell density. The green values depict yeast cell growth based on pixel counts from multiple images.


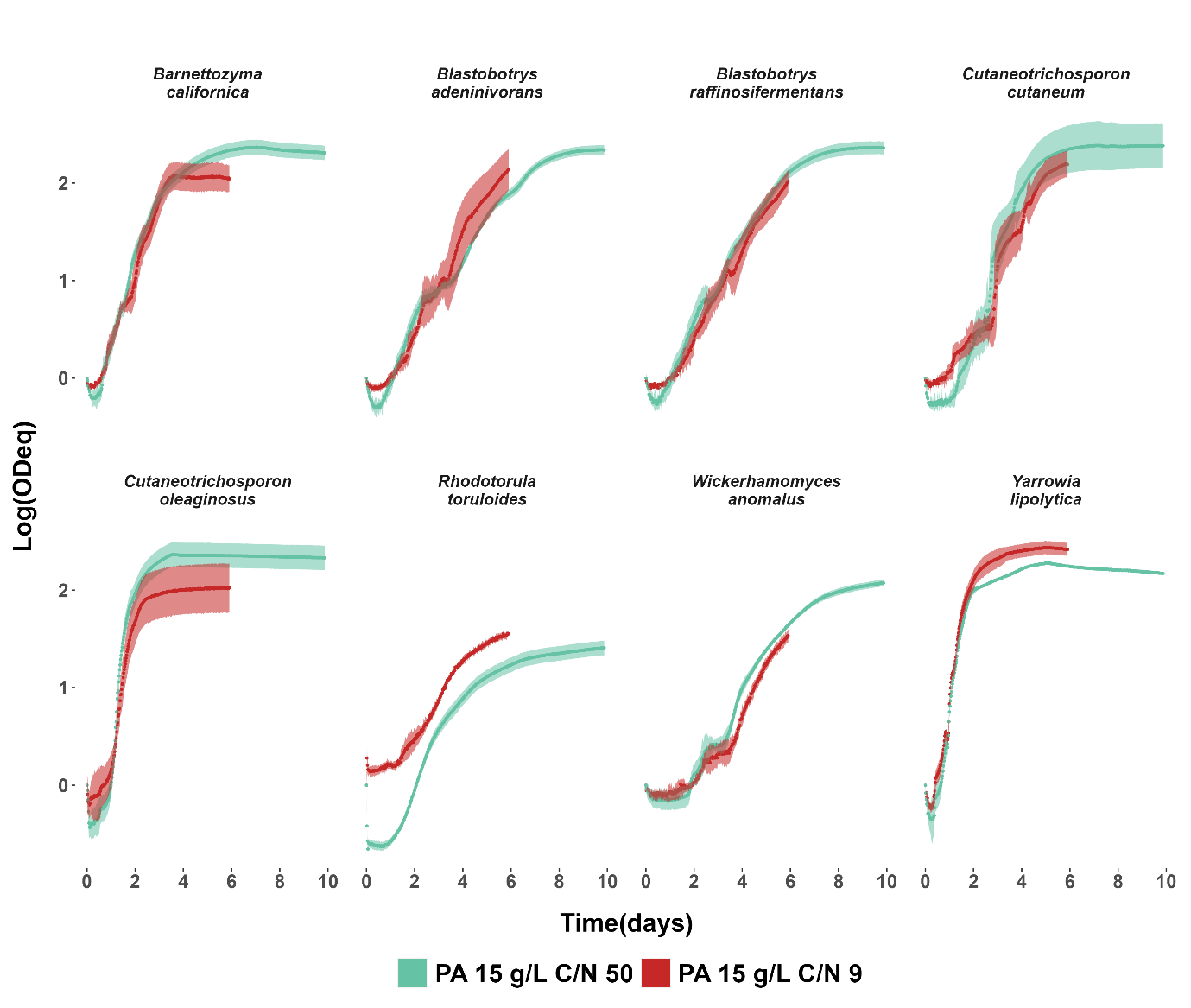


**Figure S3.** **Comparison of growth profiles in 15 g/L PA at molar C/N ratios 9 and 50**. The graphs display data obtained from a Growth Profiler in a 96-well plate system. Data are shown as mean ± standard deviation for biological triplicates. The y-axis shows the biomass formed during cultivation on a logarithmic scale using green values, considered equivalent to OD, plotted against time (days) on the x-axis. The Growth Profiler utilizes image analysis, capturing pictures from the transparent bottom of the 96-well plate every 30 minutes to measure cell density. The green values depict yeast cell growth based on pixel counts from multiple images.
